# Quantitative Biosensing Based on a Liquid Crystal Marginally Aligned by the PVA/DMOAP Composite for Optical Signal Amplification

**DOI:** 10.3390/bios12040218

**Published:** 2022-04-07

**Authors:** Tsung-Keng Chang, Mon-Juan Lee, Wei Lee

**Affiliations:** 1 College of Photonics, National Yang Ming Chiao Tung University, Tainan 711010, Taiwan; shiaubay.pt08@nycu.edu.tw; 2 National Laboratory Animal Center, National Applied Research Laboratories, Taipei 115202, Taiwan; 3 Department of Bioscience Technology, Chang Jung Christian University, Tainan 711301, Taiwan; 4 Department of Medical Science Industries, Chang Jung Christian University, Tainan 711301, Taiwan; 5 Institute of Imaging and Biomedical Photonics, College of Photonics, National Yang Ming Chiao Tung University, Tainan 711010, Taiwan

**Keywords:** optical biosensor, bovine serum albumin, cortisol, liquid crystal, tilted alignment, poly(vinyl alcohol)

## Abstract

The working principle for a liquid crystal (LC)-based biosensor relies on the disturbance in the orderly aligned LC molecules induced by analytes at the LC-aqueous or LC-solid interface to produce optical signals that can be typically observed under a polarizing optical microscope (POM). Our previous studies demonstrate that such optical response can be enhanced by imposing a weak electric field on LCs so that they are readily tilted from the homeotropic alignment in response to lower concentrations of analytes at the LC-glass interface. In this study, an alternative approach toward signal amplification is proposed by taking advantage of the marginally tilted alignment configuration without applying an electric field. The surface of glass substrates was modified with a binary aligning agent of poly(vinyl alcohol) (PVA) and dimethyloctadecyl[3-(trimethoxysilyl)propyl] ammonium chloride (DMOAP), in which the amount of PVA was fine-tuned so that the interfacing LC molecules were slightly tilted but remained virtually homeotropically aligned to yield no light leakage under the POM in the absence of an analyte. Two nematic LCs, E7 and 5CB, were each sandwiched between two parallel glass substrates coated with the PVA/DMOAP composite for the detection of bovine serum albumin (BSA), a model protein, and cortisol, a small-molecule steroid hormone. Through image analysis of the optical appearance of E7 observed under the POM, a limit of detection (LOD) of 2.5 × 10^−8^ μg/mL for BSA and that of 3 × 10^−6^ μg/mL for cortisol were deduced. Both values are significantly lower than that obtained with only DMOAP as the alignment layers, which correspond to signal amplification of more than six orders of magnitude. The new approach for signal amplification reported in this work enables analytes of a wide range of molecular weights to be detected with high sensitivity.

## 1. Introduction

Liquid crystal (LC) sensing platforms have been developed by exploiting the inherent optical and dielectric anisotropies of LCs, whose molecules are reoriented while interacting with analytes such as proteins, cancer markers, nucleic acids, metal ions, and other target molecules that are critical to the early detection of diseases and the assessment of environmental risks [1,2,3]. When the homeotropically (i.e., vertically) or homogeneously (i.e., planarly) aligned LC at the LC-aqueous or LC-solid interface was disturbed by an analyte, the interaction between LC molecules and the transmitted light was altered as well. The characteristic birefringence in LCs enables such interactions to be observed through the change in optical appearance under a polarizing optical microscope (POM) [4,5,6,7,8]. In addition to textural observation, other unique properties of LCs, particularly dielectric anisotropy and electro-optical response, are utilized to establish novel LC-based biosensors for quantitative and signal amplification purposes [5,9,10,11,12]. For biodetection at the LC-glass interface, dimethyloctadecyl[3-(trimethoxysilyl)propyl] ammonium chloride (DMOAP), a silane coupling reagent, is commonly used to maintain the vertical alignment of LC molecules through its long alkyl chain. When viewed under a POM with crossed polarizers, the vertically aligned LC gives rise to a dark appearance. As analytes accumulate on the surface of the DMOAP-modified glass substrate, the vertical anchoring strength of the LC on DMOAP is screened to a certain extent and the ordered alignment of LC is disrupted, thus inducing a dark-to-bright transition of the birefringent texture. Theoretically, analytes of higher molecular weights or concentrations are able to produce a larger area of light leakage and a more pronounced optical response.

Using bovine serum albumin (BSA) as the model protein and pristine nematic LCs such as the eutectic mixture E7 as the sensing mesogenic media, the limit of detection (LOD) for LC-based biosensors was reported to be within the order of magnitude of 10^−5^-g/mL BSA [11]. Several strategies have been developed in order to improve the LOD of LC-based detection at the LC-glass interface. On a glass surface coated with gold nanoparticles, detection of BSA by the nematic LC 4-cyano-4′-pentylbiphenyl (5CB) resulted in an improved LOD of 5 × 10^−7^ g/mL [13]. The optical response can also be enhanced by switching the sensing medium to a nematic LC with a larger birefringence (than that of E7 and 5CB) such as HDN, which gave rise to an LOD around 10^−11^ g/mL for BSA [8]. Recently, an LC–photopolymer composite was reported to amplify the optical signal of E7 so that an LOD of 3.4 × 10^−9^-g/mL BSA was achieved [11]. The LOD can be further lowered to 10^−12^ g/mL for BSA when the LC-photopolymer composite was spin-coated as a thin film in single-substrate detection [10]. In electric field-assisted signal amplification, an LOD of 10^−12^-g/mL BSA resulted when an AC voltage close to Fréedericksz’s transition threshold was applied to a negative LC [12]. While remaining vertically aligned, the weak electric field destabilized the LC molecules from the constraint of homeotropic anchoring, providing the LC with a higher potential to be tilted, leading to higher sensitivity and lower LOD. In this study, we adopt an alternative approach toward signal amplification by slightly changing the initial orientation of LC molecules in replacement of entailing an externally applied voltage.

**Figure 1:**
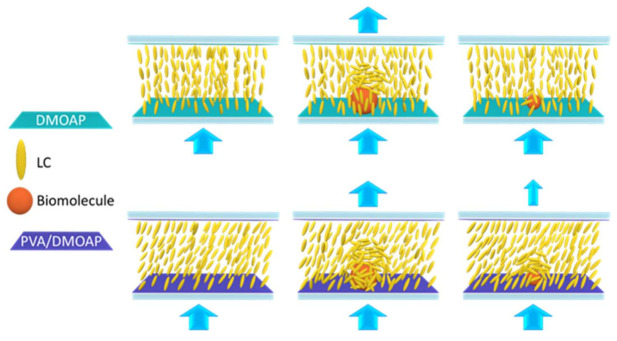
Signal amplification in LC-based biosensing through marginally tilted alignment of LCs achieved by the aligning layers of the PVA/DMOAP composite. Compared with the DMOAP-coated surface, LC molecules are in a tilted state on the PVA/DMOAP composite, which enables a greater extent of disturbance to occur in the presence of macromolecules such as BSA or small molecules such as cortisol, leading to enhanced optical signal. Blue arrows stand for the direction of light transmission.

The orientation of LCs near the LC-solid interface is highly sensitive to the composition, topography, and surface tension of the assembled alignment layer on the solid surface. Controlled photopolymerization of reactive mesogens or photoreactive monomers stabilizes LCs at pretilt angles determined through the application of an external voltage [14,15]. By altering the grafting density of an azobenzene-modified polymer on the glass surface, LCs can be induced to be either homeotropically or planarly anchored [16]. The gold nanoparticles grafted with liquid crystalline polymer with azobenzene were reported to align 5CB vertically and the transition from vertical to the random alignment of 5CB can be effectively achieved with light irradiation [17]. Moreover, the pretilt angle measured from the substrate plane is tunable by adjusting the concentration ratio of horizontal to vertical polyimides to form a mixed polyimide alignment layer, or by stacking a vertical alignment layer on a planar alignment layer [18,19]. Herein a binary planar/vertical alignment layer was produced by blending polyvinyl alcohol (PVA), a planar alignment reagent capable of creating a small pretilt angle [20,21,22], with the well-known vertical alignment surfactant DMOAP. The behavior of polyelectrolyte and surfactant systems is attributed to intermolecular interaction including electrostatic and hydrophobic interactions [23,24]. In addition to binding to the glass substrate, the negatively charged PVA [25,26] may bind to the positively charged DMOAP through electrostatic interaction in this work. As illustrated in Figure 1, due to the strong homeotropic anchoring on DMOAP, the vertical alignment of LC is disrupted only when the analyte concentration at the LC-glass interface is high enough to mask the interaction between DMOAP and LC molecules. By modifying the DMOAP monolayer with PVA to induce uneven competition between the vertical and planar surface anchoring forces, we intend to initially orient the LC in a quasi-homeotropic or marginally tilted configuration that is slightly deviated from the true homeotropic state so that the unperturbed LC orientation almost reaches the condition to cause the observable dark-to-bright transition when light leakage occurs. With the attenuated vertical anchoring, the optical response of LC in the quasi-homeotropic state would be presumably facilitated in the presence of analytes of low concentrations or molecular weights, bringing about higher detection sensitivity.

The hydrophilic polymer PVA has been used extensively in the synthesis of nanocomposite hybrid materials, and because of its biocompatibility, PVA hydrogel is developed as a potential soft tissue substitute [27,28,29]. In this work, PVA was mixed with DMOAP at various mass ratios for the preparation of mixed alignment layers on glass substrates, which were used to assemble LC cells for the detection of a macromolecule, BSA (66 kDa), and a small molecule, cortisol (362 Da), based on two nematic LCs, E7 and 5CB. The surface wettability and tilt angle were analyzed to provide evidence for the tilted state created by the PVA/DMOAP alignment. The signal enhancement realized by the PVA/DMOAP composite was assessed through image analysis of the LC optical texture observed under a POM in the crossed-polarizer scheme. Our results demonstrate that the pretilt angle of LCs is tunable by varying the PVA/DMOAP ratio without the need for an externally applied electric field, which offers a novel cost-effective approach to enhancing the optical signal and, in turn, improving the LOD of LC-based biosensors.

## 2. Materials and Methods

### 2.1. Materials

Optical glass substrates (22 mm × 18 mm × 1.1 mm) and conductive glass slides coated with indium–tin-oxide (ITO) electrodes were supplied by Ruilong Glass, Miaoli, Taiwan, and Chiptek Co., Ltd., Miaoli, Taiwan, respectively. The nematic LC E7 (with birefringence Δn = 0.2255 at 589 nm and 20 °C and clearing temperature Tc = 59 °C) and the cyanobiphenyl single compound 5CB (Δn = 0.179 at 589.3 nm and 25 °C and Tc = 35 °C) were purchased from Merck KGaA, Darmstadt, Germany, and Daily Polymer Corp., Kaohsiung, Taiwan, respectively. DMOAP (density 0.88 g/mL at 25 °C), PVA (MW ~ 30,000–50,000, 87–89% hydrolyzed), and BSA were all received from Sigma–Aldrich (St. Louis, MO, USA). Cortisol was obtained from Enzo Life Sciences (New York, NY, USA). BSA and cortisol were diluted in sterilized deionized water (DIW) to desired concentrations before the experiment.

### 2.2. Preparation of the PVA/DMOAP Composites

A 1% (*w*/*v*) aqueous solution of PVA was prepared by dissolving 1-g PVA in 100-mL distilled water under moderate stirring at 100 °C for 1 h. After cooling down to room temperature, a clear PVA solution without precipitates was achieved. For the preparation of 0.4% (*w*/*v*) DMOAP solution, 1.19 mL of a 42% (*w*/*v*) DMOAP stock solution in methanol was diluted to 100 mL with distilled water, followed by moderate stirring at room temperature for 1 h. The aqueous solutions of 1% (*w*/*v*) PVA and 0.4% (*w*/*v*) DMOAP were then mixed at various mass ratios under moderate stirring at room temperature for 1 h to obtain homogenized PVA/DMOAP mixtures for immediate substrate surface modification.

### 2.3. Surface Modification of Glass Substrates

Prior to chemical modification, the optical glass substrates were cleaned by sonication in a detergent solution for 15 min, then twice in DIW for 15 min, and finally in ethanol for an additional 15 min. After dried in an oven at 74 °C for 15 min to remove residual ethanol, the cleaned glass substrates were immersed in 0.4% (*w*/*v*) DMOAP solution, 1% (*w*/*v*) PVA solution, or various PVA/DMOAP mixtures for 30 min at room temperature. After being rinsed twice in DIW, the substrates dip-coated with DMOAP or PVA only, or with PVA/DMOAP mixtures were dried with nitrogen and baked at 100 °C for 15 min, followed by cooling at room temperature.

### 2.4. Contact Angle Measurements

The contact angle of a 5-μL water droplet on DMOAP-, PVA-, and PVA/DMOAP-coated glass substrates was determined by a Phoenix Mini contact angle analyzer (Surface Electro Optics (SEO) Co. Ltd., Suwon-si, Korea) according to the manufacturer’s instructions. Every measurement was performed within 30 s at a constant relative humidity of 40% and an ambient temperature of 25 ± 1 °C. Three substrates at each weight ratio of PVA/DMOAP composite were measured.

### 2.5. Determination of the Tilt Angle of LCs by Electrical Capacitance Measurements

ITO-glass slides were cleansed by sonication once in a detergent solution for 15 min, then twice in DIW for 15 min, and finally in ethanol for 15 min. After drying in an oven at 74 °C for 15 min to remove residual ethanol, the cleaned conductive slides were coated with PVA/DMOAP mixtures of various mass ratios for 30 min at room temperature, followed by drying with nitrogen and baking at 100 °C for 15 min. After cooling, a pair of the ITO slides covered with the same PVA/DMOAP mixture were assembled with 8-μm spacers and subsequently sealed with epoxy resin AB glue, giving rise to an assembled LC cell with a (0.5 × 0.5)-cm^2^ overlapped electrode area and a cell gap of 7.7 ± 0.5 μm. The cells were filled with E7 (dielectric anisotropy Δε = 14.3 and the parallel-component dielectric constant ε_‖_ = 19.5 at 20 °C and 1 kHz) by capillary action. To eliminate the effect of cell gap variation, we referred to our previous study [5], which reported the average tilt angle of LC, α, can be determined by the equation:

(1) $$\frac{C_{\max}-C_{0}}{C_{\max}}=\frac{\Delta \varepsilon }{\varepsilon_{\left|\right|}}\cos^{2}\alpha ,$$

where *C*_max_ and *C*_0_ are the capacitance measured at 20 and 0.5 Vrms, respectively. Electrical capacitance measurements were performed with a high-precision LCR meter (Agilent E4980A), utilizing a Tektronix arbitrary function generator (AFG 320) to supply AC voltage ranging from 0.5 to 20 Vrms at a frequency of 1 kHz in sinusoidal waveform. The corresponding conoscopic images were acquired by placing the LC cells between crossed linear polarizers and illuminated by convergent light.

### 2.6. Immobilization of Analytes and LC Cell Fabrication

In advance of the immobilization of analytes, the DMOAP- or PVA/DMOAP-coated glass substrates were rubbed unidirectionally with a rubbing machine. A 2 × 2 array was formed by depositing BSA or cortisol solutions at 3 or 1 μL/spot, respectively, on a surface-modified glass substrate. The analyte solution was incubated on the substrates at 35 °C for 3 min, and excess fluid was removed immediately by wicking with lens tissue. The LC cell was assembled by pairing two identical DMOAP- or PVA/DMOAP-coated glass substrates (separated by 8-μm ball spacers), one of which was immobilized with BSA or cortisol at a designated concentration, followed by sealing with AB glue. The empty cell was then filled with E7 or 5CB by capillary action at a temperature slightly above the clearing point. The optical texture of the LC was observed under a crossed polarized OLYMPUS BX51-P microscope (Olympus Corp., Tokyo, Japan) in the transmission mode.

### 2.7. Image Analysis with the ImageJ Software

The brightness of the optical texture at various BSA and cortisol concentrations was determined by ImageJ, a Java-based image processing software. Binary thresholding analysis was carried out on the POM images, which were converted to binary images in which black and white pixels denote the signals and background, respectively. By setting a threshold value referring to the background when the analyte was replaced by DIW, all the black pixels in the area immobilized with biomolecules were summated by ImageJ. At least three repeated experiments were executed to calculate the average and standard deviation of the relative intensity at each concentration of the analyte in the E7- and 5CB-based sensing platform. The brightness of the optical texture at the lowest concentration of the analyte, which can be distinguished from that of the DIW-only blank control when inspected under POM, was determined in at least seven replicate experiments to calculate the LOD.

## 3. Results and Discussion

### 3.1. Optical Texture of E7 on PVA/DMOAP-Coated Glass Substrates

**Figure 2:**
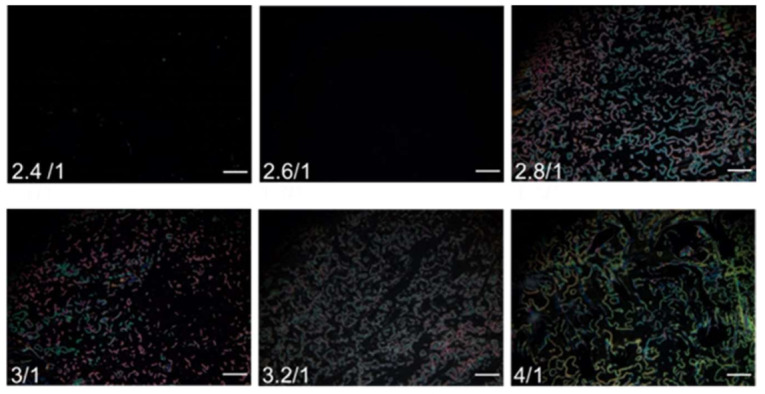
Optical texture of E7 on glass substrates coated with the PVA/DMOAP composite. The nematic LC E7 was sandwiched between two glass substrates coated with PVA/DMOAP mixtures of various mass ratios (2.4:1, 2.6:1, 2.8:1, 3:1, 3.2:1, and 4:1). Scale bar: 500 μm.

To maintain the vertical anchoring of LC in the absence of analytes, it is necessary to optimize the amount of PVA in the PVA/DMOAP mixture for surface modification. The orientation of E7 sandwiched between two PVA/DMOAP-coated substrates was examined under the POM. At PVA/DMOAP mass ratios of 2.4:1 and 2.6:1, no light leakage was found in the optical texture of E7, inferring that although the vertical anchoring strength of DMOAP was presumably weakened by PVA, its amount was insufficient to essentially alter the alignment of LC to scatter the transmitted light when observed under the POM (Figure 2). However, when the PVA/DMOAP mass ratio was increased to 2.8:1 and higher, the textural appearance of E7 became bright, indicating that the amount of PVA has reached a threshold where the vertical anchoring strength on DMOAP was disrupted so that the LC molecules were no longer homeotropically aligned. A PVA/DMOAP mass ratio of 2.6:1 was therefore adopted in the following biosensing studies to ensure that the LC molecules remained in the quasi-homeotropic state in the absence of analytes.

### 3.2. Contact Angle Measurements on DMOAP-, PVA- and PVA/DMOAP-Coated Glass Substrates

**Figure 3:**
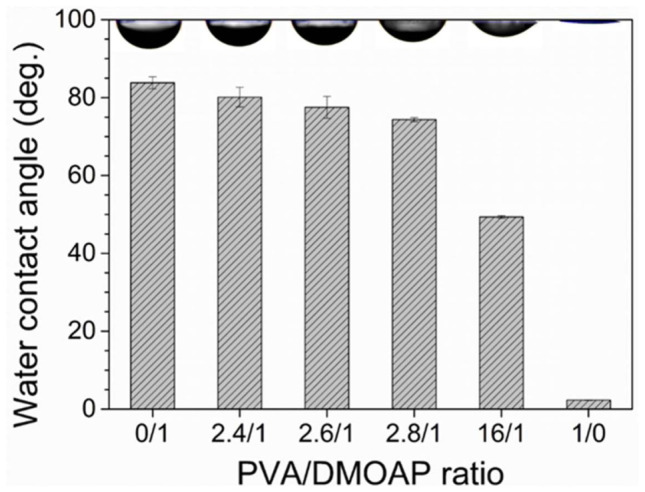
Water contact angle measured on glass substrates coated with the PVA/DMOAP composite. Glass substrates were coated with PVA/DMOAP mixtures of various mass ratios (0:1, 2.4:1, 2.6:1, 2.8:1, 16:1 and 1:0) and subjected to water contact angle measurements. Representative images of the water droplet at each PVA/DMOAP mass ratio are shown at the top of the chart. Error bars represent standard deviations calculated from at least three independent measurements.

The wettability of the DMOAP-, PVA- and PVA/DMOAP-coated glass surface was compared by means of contact angle measurements. As shown in Figure 3, in contrast to the DMOAP monolayer that was less hydrophilic yielding a water contact angle of 84°, the superhydrophilic PVA monolayer gave rise to a contact angle of 2°, which was consistent with previous studies [7,30]. For the glass surface modified with the aligning PVA/DMOAP composite, a decrease in contact angle was observed when the PVA/DMOAP ratio was increased from 2.4:1 to 16:1. This finding suggests that the hydrophilicity of the glass surface can be enhanced by increasing the proportion of PVA in the binary PVA/DMOAP mixture. By applying Wenzel’s equation that correlates surface roughness to contact angles smaller than 90°,

(2) cosθ*=γcosθ,

where θ* and *θ* represent contact angles of the PVA/DMOAP- and DMOAP-coated surfaces, respectively, and *γ* is the roughness factor [31], the roughness of the PVA/DMOAP-coated surface compared with that coated with DMOAP alone can be assessed. Because PVA resulted in a decrease in θ* such that θ* < *θ* and *γ* > 1, surface roughness was increased in the presence of PVA partially constituting the aligning layer.

### 3.3. Tilt Angle of LCs on Substrates Modified with the PVA/DMOAP Composite

**Figure 4:**
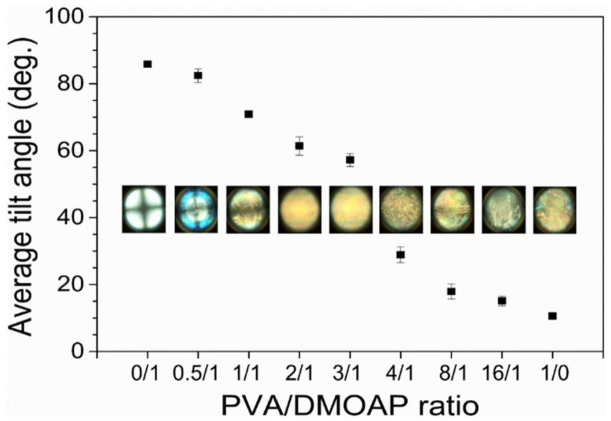
Average tilt angle of E7 as determined by capacitance measurements. The nematic LC E7 was sandwiched between two ITO-coated glass substrates covered with the PVA/DMOAP aligning agent. The average tilt angle was calculated by Equation (1) and plotted against the PVA/DMOAP mass ratio. Error bars represent standard deviations calculated from at least three independent measurements. Corresponding conoscopic micrographs at distinct PVA/DMOAP mass ratios are displayed for comparison.

The average tilt angle of E7 on the PVA/DMOAP-coated glass substrate was specified by capacitance measurements and plotted against the PVA/DMOAP mass ratio as shown in Figure 4. Without PVA (PVA/DMOAP ratio 0:1 in Figure 4), the E7 molecules on the DMOAP-coated surface exhibited an average tilt angle of 85.85 ± 0.98°, indicating that the LC molecules were aligned homeotropically. As the content of PVA increased, the average tilt angle decreased until reaching a minimum of 10.6 ± 0.13° at a PVA/DMOAP ratio of 1:0, in agreement with the planar alignment caused by the aligning agent PVA. Corresponding conoscopic images suggest that E7 was homeotropically aligned at a PVA/DMOAP ratio of 0:1, whereas the vertical alignment strength was slightly attenuated when the PVA/DMOAP ratio was increased to 0.5:1 (α = 82.13 ± 0.12°), consistent with the results of the average tilt angle (Figure 4). The orientation of E7 molecules deviated from homeotropic alignment at PVA/DMOAP ratios of 1:1 and above as the Maltese cross was no longer visible in the conoscopic interference pattern. Note that capacitance measurements, as well as conoscopic analysis, were accomplished on LC cells composed of ITO-coated glass to exhibit different surface conditions from those of optical glass. The absolute value of the average tilt angle presented in Figure 4, therefore, does not necessarily correlate with the change in optical texture and contact angle observed on optical glass, as shown in Figure 2 and Figure 3. Nevertheless, results in Figure 4 provide strong evidence that the tilt angle of LC is tunable by simply adjusting the PVA proportion in the PVA/DMOAP aligning composite.

### 3.4. Detection of BSA on the PVA/DMOAP Composite

**Figure 5:**
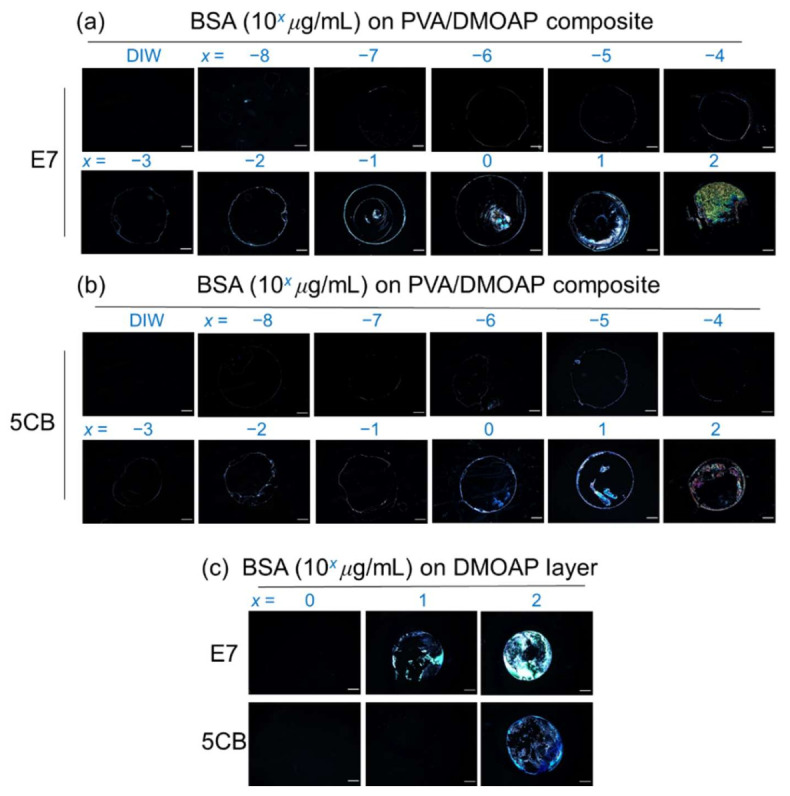
Optical texture of E7 and 5CB at various concentrations of BSA on glass substrates coated with the PVA/DMOAP composite. Aqueous solutions of BSA at various concentrations (10^−8^ to 10^2^ μg/mL) were immobilized on PVA/DMOAP-coated glass substrates for LC-based detection with (**a**) E7 and (**b**) 5CB as the sensing media. (**c**) A parallel study on DMOAP-coated glass substrates was undertaken with a BSA concentration range of 10^0^–10^2^ μg/mL. Scale bar: 500 μm.

The PVA/DMOAP composite at a PVA/DMOAP ratio of 2.6:1 was employed in the detection of BSA, a protein standard with a molecular weight of 66 kDa (Figure 5). When observed under a POM, the lowest BSA concentration at which the optical response of E7 can be distinguished from the dark optical appearance in the presence of DIW was merely 10^−8^ μg/mL, and as expected, the brightness of the optical texture increased with increasing BSA concentration from 10^−8^ to 10^2^ μg/mL (Figure 5a). The “coffee ring” seen in several of the POM images was due to uneven distribution of the solute (BSA) during solvent (water) evaporation, which was slower in the central liquid-vapor interface than on the rim of the droplet of BSA solution, leading to a radially outward capillary flow of BSA to the boundary area [32]. Compared with 5CB within the same BSA concentration range, the higher level of light leakage in E7 caused by BSA implies that E7 is more sensitive than 5CB to the change in the number of analytes at the LC-glass interface (Figure 5a,b). This is supported by our previous finding that LC of larger birefringence (E7 in this study) enhances signal amplification and sensitivity in biosensing by achieving higher transmitted light intensity (stemming from drastically scattered light owing to a more severe index mismatch in the LC bulk) between cross polarizers [8]. In comparison with E7, the narrow nematic temperature range of 5CB (23.6 to 35.2 ℃ for 5CB vs. −10 to 59 °C for E7) connotes that 5CB is prone to phase transition at ambient temperature. Besides, the effective birefringence of 5CB was also reported to be more temperature-sensitive, especially when approaching the nematic-to-isotropic transition temperature (Tc) [33,34]. Consequently, 5CB may not be a stable or suitable sensing material for the development of fast-screening or point-of-care devices desired to be accustomed to various climate zones unless in a temperature-controlled environment.

When detected on glass substrates coated only with DMOAP, the lowest detectable BSA concentration was significantly higher compared with that in the presence of binary PVA/DMOAP alignment layers for both E7 and 5CB, which was on the orders of magnitude of 10 and 100 μg/mL (Figure 5c), respectively. It is thus evident from these results that biosensing with LC in the marginally tilted state induced by the PVA/DMOAP blend led to the enhanced optical response. It should be noted that at 10^2^-μg/mL BSA, the optical texture of E7 on DMOAP-coated glass substrate was brighter than those interfaced with the PVA/DMOAP composite (Figure 5c). This can be explained by the reduced contact angle on the PVA/DMOAP surface to cause less BSA adsorption and by the electrostatic repulsion between the negatively charged PVA and BSA [35,36].

**Figure 6:**
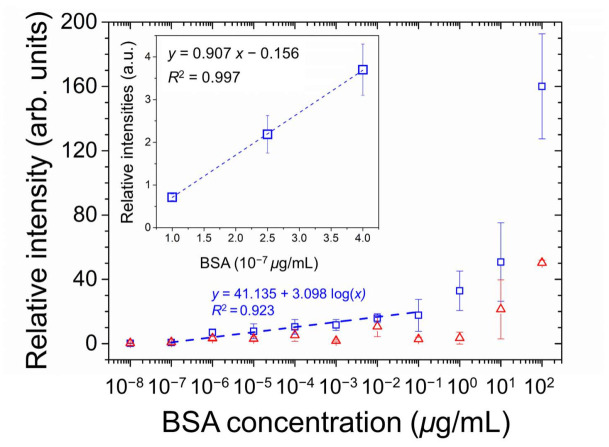
Quantitative analysis of the optical responses of E7 (open squares) and 5CB (open triangles) at various concentrations of BSA on glass substrates coated with the PVA/DMOAP composite. The relative intensity of the brightness of the optical texture in Figure 5a,b were determined by the image processing program ImageJ and plotted against the logarithmic BSA concentration. The linear curve was illustrated in the inset to calculate the LOD. Error bars represent standard deviations calculated from at least three independent experiments. The dotted line represents the fitted regression line, with the determination coefficient R^2^ given in the legend.

The optical responses of E7 and 5CB were quantitated through image analysis to calculate the relative intensity of the optical texture, which is plotted against the logarithm of BSA concentration as shown in Figure 6. Linear regression analysis of E7 sensing performance was attempted on the semi-logarithmic plot in the BSA concentration range of 10^−7^ to 10^−1^ μg/mL, yielding a correlation coefficient R^2^ of 0.923. The LOD was calculated by the following equation:

(3) $$\mathrm{LOD}=\frac{3s}{m},$$

where *s* is the standard deviation of relative intensity at the lowest BSA concentration whose value is significantly higher than that at 0-g/mL BSA, and m is the slope of the linear regression line [37]. The relative intensity at the lowest concentration of BSA (10^−7^ μg/mL) was significantly higher than that of the DIW-only blank control. According to the inset of Figure 6, the LOD was calculated from the results of linear regression of the relative intensities at 1, 2.5, and 4 × 10^−7^-μg/mL BSA. The deduced LOD is 2.5 × 10^−8^-μg/mL BSA, which was nine orders of magnitude lower than that of LC-based biodetection exploiting an alignment layer consisting only of DMOAP.

### 3.5. Detection of Cortisol on the PVA/DMOAP Composite

**Figure 7:**
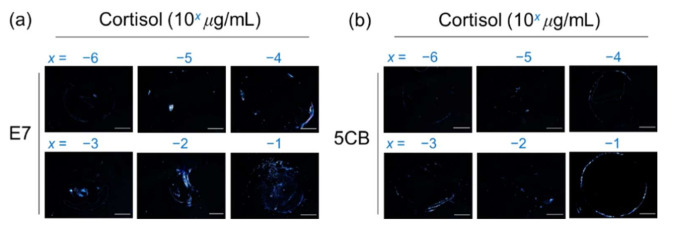
Optical texture of (**a**) E7 and (**b**) 5CB at various concentrations of cortisol on glass substrates coated with the PVA/DMOAP composite. Aqueous solutions of cortisol at various concentrations (10^−6^–10^−1^ μg/mL) were immobilized on PVA/DMOAP-coated glass substrates for LC-based detection with E7 or 5CB as the sensing medium. Scale bar: 500 μm.

**Figure 8:**
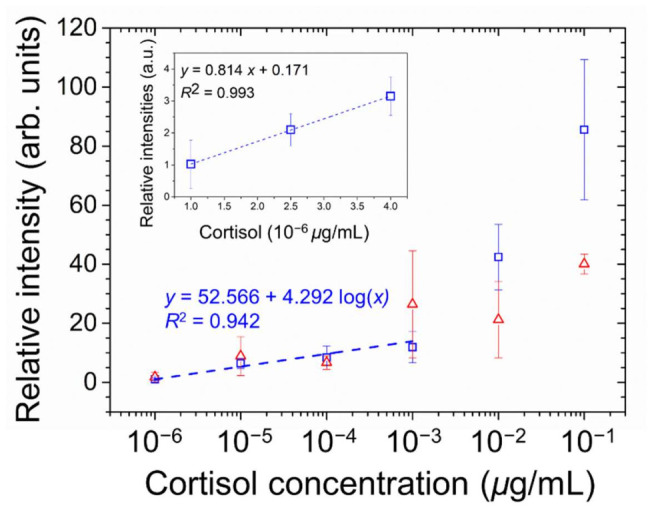
Quantitative analysis of the optical responses of E7 (open squares) and 5CB (open triangles) at various concentrations of cortisol on glass substrates coated with the PVA/DMOAP composite. The relative intensity of the brightness of the optical texture in Figure 7a,b were determined by the ImageJ software and plotted against the logarithm of cortisol concentration. The linear curve was illustrated in the inset to calculate the LOD. Error bars represent standard deviations calculated from at least three independent experiments. The dotted curve denotes the fitted regression line, with the coefficient of determination, R^2^, shown in the legend.

Cortisol, a glucocorticoid and a stress hormone secreted by the adrenal gland with a molecular weight of 362 Da, was applied as a small-molecule analyte to further demonstrate the signal amplification effect based on the marginally tilted alignment of E7 and 5CB realized by the PVA/DMOAP composite. Daily fluctuation of the level of cortisol in human serum is well known, and the average concentration of serum cortisol in the morning, which appears much higher than that in the afternoon, was reported to be around 0.1 μg/mL [38]. Accordingly, a maximal concentration of 0.1-μg/mL cortisol was arbitrarily chosen for the proposed LC-based detection. For both E7- and 5CB-engaged sensing platforms at cortisol concentrations ranging between 10^−6^ and 10^−1^ μg/mL, the brightness of the optical texture of LCs increased with the increasing concentration of analytes on the PVA/DMOAP composite layer (Figure 7a,b), whereas no optical response was detected on the DMOAP-coated surface even for the highest cortisol concentration applied (data not shown). The relative intensity of the LC optical texture was plotted as a function of the cortisol concentration as shown in Figure 8. The relative intensity at the lowest concentration of cortisol (10^−6^ μg/mL) was significantly higher than that of the DIW-only blank control. According to the inset of Figure 8, the LOD was calculated from the results of linear regression of the relative intensities at 1, 2.5, and 4 × 10^−6^-μg/mL cortisol. The calculated LOD was 3 × 10^−6^-μg/mL cortisol for characterizing the sensing performance of the E7 biosensors. These results demonstrate that, in addition to macromolecules such as proteins, the sensitive detection relying on the quasi-homeotropic alignment of LCs can be extended to label-free biodetection and quantitation of small molecules with molecular weights lower than 500 Da.

## 4. Conclusions

In this study, two analytes, BSA (66 kDa) and cortisol (362 Da), of extreme discrepancy in molecular weight were detected at concentrations of 10^−7^ to 10^−6^ μg/mL by an LC-based optical biosensor constructed with two glass substrates coated with hybrid PVA/DMOAP aligning layers. Dramatic amplification of optical signal resulted from the initialization of marginally tilted alignment of LC molecules without the need to apply an external electric field [12]. By slightly weakening the vertical anchoring strength stemming from the typical aligning agent DMOAP through the incorporation of PVA, the detection limit of the LC-based biosensing platform substantially outperformed that of the counterpart with pure DMOAP coatings. The analytes studied in the present work represent a wide range of molecular weights. Because the pretilt angle of LC can be readily tunable by adjusting the composition in the binary PVA/DMOAP aligning agent, surface functionalization with the binary mixture for LC alignment is a simple and cost-effective approach for signal amplification that can be easily applied to analytes of various molecular weights ranging from small molecules to proteins.

## Data Availability

Data underlying the results presented in this paper are not publicly available at this time but may be obtained from the authors upon reasonable request.
